# Medical Organisation in German S.W. Africa: The Transport Problem

**Published:** 1915-06-26

**Authors:** 


### WITH THE SOUTH AFRICAN MEDICAL CORPS.

### Medical Organisation in German S.W. Africa.

### THE TRANSPORT PROBLEM.

The following interesting passages occur in a letter just received from a medical officer with the Southern Force that has penetrated German South-West Africa:—

#### A Small Brigade.

I am glad to say we have been sent up at last, and after a week at —— set out to join our brigade. Our first trek lasted eight days, and was pretty trying whilst it lasted. We really form a small brigade in ourselves. We have six ordinary wagons, six ambulance wagons, two Scotch carts, two motor-ambulances and two water-carts, 180 mules, eighteen horses, sixty-four men, and about forty natives.

Each regiment has its medical officer, whilst we travel immediately behind the brigade, which consists of, roughly, 4,000 men.

Any case more serious than a headache, or one which is lying down for more than twenty-four hours, is sent back to us by the regimental medical officer. If we have more than twenty-four such cases, we have to pitch tents and become "immobile." But we, on the other hand, clear out cases to base hospitals further back still, and so endeavour to keep our "mobility."

#### The Invaluable Ass.

We ourselves have penetrated some miles within German territory, whilst our brigade outposts are not far from the —— railway. The border is being thoroughly cleared until we move further forward. Our great difficulty, of course, is supplies. It is a fearful trek up from ——. Supplies take about ten days to get up here, although the whole brigade is not up. The position has been relieved to a certain extent by our capturing 2,000 cattle and 11,000 sheep. But the greatest difficulty is to supply food for the animals. Thus an ox- or mule-wagon will have eaten half its load before it arrives here. The only animal that has distinguished itself in this part of the world is the humble donkey. It only wants 5 lbs. of mealies per day, and a span of sixteen can take a load of 4,000 lbs. twenty miles a day. The saying here now is that it is no insult to be called an ass!

#### Medical Outfit.

All the regimental staffs think our huge outfit absurd and cumbersome, but we soon were able to make them look absurd and feel thankful. One regiment had carried a case of appendicitis for four days, and it was practically dying when we got here. We immediately pitched our operating tent, took out our large panniers, and had operated within a few hours of our arrival. We have undoubtedly saved his life, and no one would have been able to do so without an equipment.

We carry whiskey, brandy, champagne, port, Bovril, cornflour, milk, and all sorts of other medical comforts and necessaries. There is no doubt that the Government is sparing no expense over the campaign, although there is a good deal of leakage and bungling over the administration. Ours seems an expensive unit to equip and keep up. One felt what an expense to the Government we were during one week at ——, and during the week's trek up here, when the sum total of our medical work was the extraction of two teeth, and one of them was broken! However, we have saved one life already, and once heavy fighting begins, I am certain we shall save many more.

You can imagine the relief to a poor devil who has been roughing it for some weeks, and then to become ill under the same conditions, to get into our large marquee with clean pyjamas, hot nourishing food, a soft bed, and to be waited on hand and foot.

#### A Strange Contrast.

We live here among strange contrasts; whilst we can get barely sufficient supplies to keep us going, at the same time we get all the world's news probably before you get it. We have a wireless here, and at certain intervals during the day Slangkop sends out its budgets. It takes us about ten hours to communicate with a regiment some miles from here, yet we hear the European news within a few hours.

#### The German View.

I think there is no doubt that an expedition into German West Africa from the —— side was put out of court by the Germans because of the difficulties of transport. The unusual rains have made it possible, although very slow and laborious. The Germans left —— in a hurry, and at ——, where we now are, troops disturbed them just as they were sitting down to a meal. They left it, but scribbled on the walls in English and German, "This food is poisoned." The tale goes that some was sent out to a Hottentot, who survived the test, and a general tuck-in ensued.

We will probably be here some days; meanwhile flies, sand, heat, and mosquitoes are our daily portion.

